# Rotating Polarization Magnetometry

**DOI:** 10.3390/s25092682

**Published:** 2025-04-24

**Authors:** Szymon Pustelny, Przemysław Włodarczyk

**Affiliations:** 1 Institute of Physics, Jagiellonian University in Kraków, Łojasiewicza 11, 30-348 Kraków, Poland; 2 Department of Physics, Harvard University, Cambridge, MA 02138, USA

**Keywords:** optical atomic magnetometry, optically pumped magnetometers, nonlinear magneto-optical rotation, light with continuously rotating linear polarization, amplitude-modulated light, alignment-to-orientation conversion

## Abstract

Precise magnetometry is vital in numerous scientific and technological applications. At the forefront of sensitivity, optical atomic magnetometry, particularly techniques utilizing nonlinear magneto-optical rotation (NMOR), enables ultraprecise measurements across a broad field range. Despite their potential, these techniques reportedly lose sensitivity in higher magnetic fields, which is attributed to the alignment-to-orientation conversion (AOC) process. In our study, we utilized light with continuously rotating linear polarization to avoid the AOC, which produced robust optical signals and achieving high magnetometric sensitivity over a dynamic range nearly three times greater than Earth’s magnetic field. We demonstrated that employing rotating polarization surpasses other NMOR techniques that use modulated light. Our findings also indicate that the previously observed signal deterioration was not due to the AOC, suggesting an alternative cause for this decline.

## 1. Introduction

Optical magnetometry belongs to one of the most mature quantum technologies to date [1,2,3,4,5,6,7,8,9,10,11,12,13,14]. Modern optical atomic magnetometers (OAMs) offer a magnetic field sensitivity often exceeding 10 fT/Hz^1/2^, with the only sensitivity-wise competitors being bulky and high-maintenance superconducting quantum interference devices (SQUIDs). This remarkable sensitivity, combined with technical simplicity and low operating costs, has led to a wide range of applications, from biomagnetic field measurements [15,16,17,18,19], surveys of natural resources [20,21], or magnetic induction tomography [22,23] to zero-field nuclear magnetic resonance detection [24,25,26] and searches for ultra-light dark matter [27,28,29,30] (for more information, see, for example, Ref. [2] and references therein).

The highest sensitivity of OAMs is achievable in very weak magnetic fields, necessitating effective shielding against external and uncontrolled magnetic fields. Operation in stronger fields, such as Earth’s magnetic field, requires the implementation of solutions that can, at least partially, capitalize on the low-field sensitivity. For example, the stronger field could be compensated for by using a set of calibrated magnetic field coils to bring the operating point of the magnetometer back to zero [31,32,33,34]. By actively bringing the field to zero, one can (nominally) maintain the zero-field sensitivity, additionally obtaining directional sensitivity through magnetic field modulation [31,34]. Unfortunately, due to the limited stability of current sources and the inhomogeneities induced by the compensation of the magnetic fields, these methods still suffer from sensitivity losses.

An alternative technique that has recently regained interest is based on the observation of the magnetization decay of optically polarized atomic vapors [35,36,37,38,39]. By analyzing a time-dependent signal arising after the pulsed polarization of the atoms, one determines the spin precession frequency, and hence, the magnetic field to which the atoms are exposed to. Despite its utility, this approach does have some drawbacks, including (in some cases) a relatively involved signal analysis and the inability to perform continuous measurements.

The third group of methods is based on modulating certain physical parameters of the system to enhance the sensitivity in high fields. A notable example of this approach is the so-called M_*x*_ magnetometry [7,40,41,42], which is based on the application of an external oscillating magnetic field and the observation of light transmission through the magneto-optically active medium. By an appropriate choice of the oscillating field frequency, one can resonantly excite spin precession, generating the strongest dynamic response of the medium. In turn, by tracking the resonance position, one can monitor the magnetic field strength. While in the M_*x*_ magnetometers, circularly polarized light is typically used, similar measurements can be achieved with linearly polarized light [43]. In this case, one can either detect static or oscillating magnetic fields by controlling the other field frequency or magnitude. The drawback of these techniques, however, is the necessity of applying an external field, which, in the case of multiple sensors, for example, for biomagnetic field imaging [15], leads to cross-talk between the devices. Alternatively, the additional field can be replaced by the modulation of interacting light parameters [2]. This was already demonstrated in the first Bell–Bloom magnetometer [44], where the pumping light intensity was modulated, giving rise to the so-called high-frequency resonance arising when the modulation frequency coincides with the magnetization precession (Larmor) frequency of atoms. This technique was further developed in the scope of magnetometric techniques that explore nonlinear magneto-optical rotation (NMOR), sometimes also called the nonlinear Faraday effect (when the light propagation and magnetic field directions coincide) [3], where both the frequency [45,46,47] and amplitude [8,48,49,50] modulation were used to measure stronger magnetic fields.

A problematic feature of NMOR magnetometers is the deterioration of the amplitude of high-frequency resonances [51], and hence, the magnetic field sensitivity with an increasing magnetic field. It is known that this deterioration and broadening of the resonance can be attributed to the splitting observed in the NMOR resonance due to the nonlinear Zeeman effect, where resonances associated with different magnetic sublevels are unequally shifted [46,52]. Additionally, it has been postulated that this effect may also originate from the so-called alignment-to-orientation conversion (AOC) [53]. This effect arises when the electric field of the light is not parallel to the atomic polarization and it consists of the transfer of the atomic polarization (alignment) that is detectable in NMOR into atomic polarization (orientation) that is undetectable with this method. It should be noted, however, that the process of AOC conversion arises when there is a non-zero angle between the light and atomic polarizations, i.e., it disappears when they are parallel.

In this work, we present the application of light with rotating linear polarization for optical magnetometry. By precisely matching the atomic Larmor frequency with the frequency of polarization rotation, we generated dynamic polarization of the medium. This dynamic atomic polarization led to the polarization rotation of the second initially unmodulated light beam. The parameters of the induced NMOR resonances were measured against factors such as the light intensity and tuning. Since the orientations of the atoms and light polarization remained parallel at all times, i.e., the AOC did not arise, this arrangement allowed us to test the role of the effect in the deterioration of magnetometric sensitivity in higher fields. Specifically, we conducted a comparative analysis between the rotating polarization (RotPol) and amplitude-modulated NMOR (AMOR) methods, as the AOC should play a detrimental role in the latter case. Through these investigations, we aimed to provide a direct comparison of the two techniques for magnetic field measurements, which offered valuable insights into their respective sensitivities and applications.

## 2. Experimental Setup

**Figure 1:**
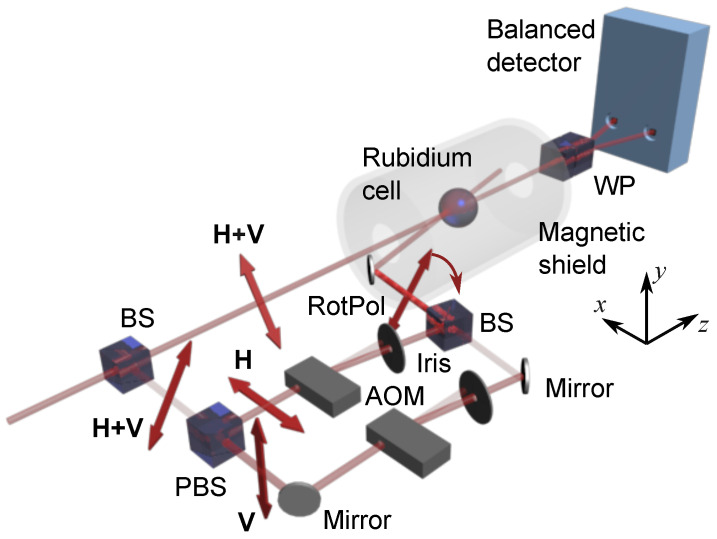
Schematic of the experimental setup used for the field measurements. BS stands for a beam splitter, PBS is a polarization beam splitter, WP is a Wollaston prism, AOM is an acousto-optic modulator, **H** and **V** indicate horizontal and vertical linear polarizations, and **H + V** is the polarization oriented at 45°.

The scheme of the experimental system used in our measurements is shown in Figure 1.

The heart of the system was a spherical vapor cell of 3 cm in diameter that contained an isotopically enriched sample of ^87^Rb at a slightly elevated temperature of 45 °C. To prolong the atomic polarization lifetime, the cell walls were coated with a special anti-relaxation layer, which extended the lifetime to approximately 30 ms (with polarization preserved for up to 4000 wall collisions). The cell was placed inside a magnetic shield made of three layers of mu-metal, which enabled magnetic field shielding at a level of 10^4^ (TwinLeaf LLC, Plainsboro, NJ, USA). A set of magnetic field coils was placed inside the shield to compensate for the residual fields and generate a leading field along the probe light propagation direction.

The cell was illuminated with two light beams: the probe and the pump. If not stated otherwise, both beams were extracted from a diode laser (Toptica DL Pro, Toptica, Munich, Germany) with a wavelength tuned to the rubidium D_1_ line (795 nm). The linearly polarized light emitted from the laser was split using a beam splitter (BS) and directed into two parts of the experimental setup. The first fraction of the beam was directed into a system generating light with continuously rotating linear polarization. While this system was described in detail in Ref. [54], here we recall some of its crucial elements. In general, the system was based on a Mach–Zehnder interferometer, in which two orthogonal linear polarizations were directed into separate arms of the device using a polarization beam splitter (PBS). In each arm of the interferometer, an acousto-optic modulator (AOM), which was optimized for first-order diffraction, was used (Isomet LTD, Torfaen, UK). Both AOMs were driven with acoustic waves of roughly 80 MHz that were extracted from the same generator. While one of the modulators was directly driven by the generator signal, the other driving signal was additionally modulated using a single-sideband modulator, which allowed us to coherently shift its frequency by a much lower frequency $\nu_{m}$. The beams directed into the first order of diffraction of both AOMs were then recombined using a beam splitter. The superposition of the orthogonally polarized beams frequency-shifted by $\nu_{m}$ resulted in the generation of light with continuously rotating polarization. It should be stressed that, as discussed in Ref. [49], light with continuously rotating linear polarization cannot be generated either with a rotating half-wave plate or with the combination of an electro-optic modulator and birefringent elements. It is also noteworthy that a spatial interference pattern of the light that left the interferometer did not affect the optical pumping in our experimental arrangement, where a paraffin-coated cell was used (for more details, see Ref. [55]). This light was then used to optically pump the rubidium atoms.

The unmodulated part of the original beam was used to illuminate the vapor to probe its spin polarization. After passing through the cell, the polarization of this probe beam was detected using a balanced polarimeter, which consisted of a Wollaston prism (WP) that split the light into orthogonal polarization components and a balanced detector (Thorlabs, Newton, NJ, USA). The difference signal of the detector was proportional to the polarization rotation of the light. The balanced polarimeter output signal was demodulated at the frequency $\nu_{m}$. This allowed the measurements of the NMOR signal versus different experimental parameters, such as detuning and both light beams’ intensities.

A fraction of the probe beam, which was split out of the probe prior to entering the shield, was used for stabilization and monitoring of the light wavelength. The monitoring was achieved through absorption spectroscopy, while stabilization was implemented using a dichroic atomic vapor laser lock that exploited a microscopic vapor cell. In all measurements, except those that involved wavelength dependence, the light was slightly detuned toward the low-frequency wing of the Doppler-broadened $F=2\to F^{\prime }=1$ transition.

## 3. Results

### 3.1. NMOR Signals

**Figure 2:**
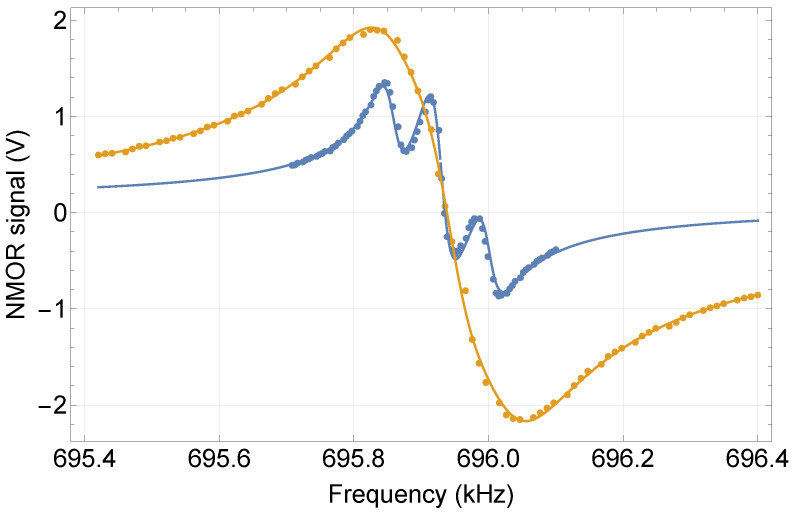
RotPol signals measured at a magnetic field of 100 μT versus the polarization rotation frequency for pump powers of 10 μW (blue) and 300 μW (orange). The experimental signals (points) were fitted with the triple-Lorentzian profile given by Equation (1) (solid lines). The signals were measured with a probe power of 10 μW.

Figure 2 shows the signal recorded with the RotPol at roughly 100 μT, i.e., a field three times larger than Earth’s magnetic field in our lab. To investigate the role of the pumped-light power, the signals were measured for both weak (10 μW) and strong (300 μW) pump powers.

At low pump powers, the observed signal clearly consisted of three resonances. This triple structure was a consequence of the nonlinear Zeeman effect, which lifted the degeneracy of the magnetic sublevel splitting beyond the resonance widths. At higher powers, the amplitudes of the resonances increased, which resulted from more efficient optical pumping, but the composite resonances were power-broadened. In particular, at 300 μW, the broadening of the individual resonances exceeded their width such that the signal manifested as a single resonance.

To extract the quantitative information about the signals, a triple-Lorentzian profile was fitted to the experimental data (solid lines in Figure 2):

(1) $$f=\left(\frac{A}{\frac{\nu \;-\;\nu_{0}}{\gamma }+i}+\frac{A_{1}}{\frac{\nu \;-\;\nu_{0}\;+\;\nu_{0}^{2}/\Delta_{\mathrm{HF}}}{\gamma_{1}}+i}+\frac{A_{1}}{\frac{\nu \;-\;\nu_{0}\;-\;\nu_{0}^{2}/\Delta_{\mathrm{HF}}}{\gamma_{1}}+i}\right)e^{i\phi },$$

where *A* and $A_{1}$ are the amplitudes of the composite resonances; γ and $\gamma_{1}$ are their widths; $\nu_{0}$ is the Larmor frequency, and hence, the position of the central resonance; ϕ is the global phase; and $\Delta_{\mathrm{HF}}$ is the rubidium-87 hyperfine frequency [56]. The triple character of the signal originated from the three pairs of magnetic sublevels, each of which was responsible for the appearance of one resonance. Since the measurements were performed in stronger magnetic fields, the positions of two resonances were shifted by a quadratic Zeeman contribution (toward lower and higher frequencies), which gave rise to satellite resonances (for more details, see Ref. [46]). Note that in the fitting, the satellite resonances had the same amplitude and width, which, however, were different from those of the central resonance. Fitting the signals enabled a comprehensive investigation of the resonance parameters and successively determined the sensitivity of the magnetic field measurements as a function of the experimental parameters, such as the pump and probe powers and tunings.

### 3.2. NMOR with Rotating Polarization vs. Amplitude-Modulated Light

One of the main questions of this study was whether the RotPol approach offers superior performance compared with the method utilizing AM light. To investigate this, we measured NMOR signals using two schemes: one employed rotating polarization and the other used amplitude modulation of the pump light. When placing the high-quality polarizer after the interferometer, AM light with 100% modulation at twice the polarization rotation frequency was achieved, which enabled AMOR measurements [48].

To ensure a fair comparison between the RotPol and AMOR, an important question arose: should the comparison be based on signals with the same average light intensity or the same amplitude of modulation of the pump (bearing in mind the two-fold difference between the average pump power in these scenarios)? Given the validity of arguments for both cases, we explored and compared a RotPol signal with AMOR signals measured for both cases.

**Figure 3:**
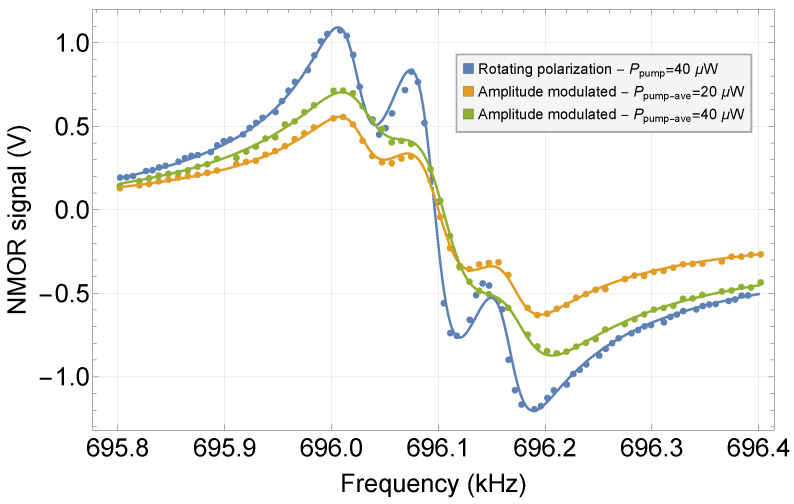
Comparison of the NMOR signals measured with the RotPol and AM lights. The parameters extracted based on triple-Lorentzian fits are given in Table 1.

Figure 3 shows representative RotPol and AMOR signals measured in the same magnetic field (roughly Earth’s magnetic field), with identical pump and probe tunings and the same probe power.

The results highlight a distinct trend: stronger signals were observed when utilizing the rotating polarization pump. In fact, the RotPol signal amplitude was roughly two times larger than the AMOR signal amplitudes (see Table 1). Moreover, in both of the AMOR cases, the widths of the resonances were notably larger. This effect originated from additional relaxation that arose due to the pump; as the pump intensity followed a sinusoidal modulation, there was a finite probability of repumping the atoms that were already polarized by the light. Since newly created polarization deviates in orientation from the original one, this process deteriorated the overall transverse polarization of the atoms, which acted as an additional relaxation. As might be expected, this effect was more pronounced at a stronger pump power (Table 1). Specifically, while the AMOR signal amplitude underwent a change of approximately 25% when going from 10 μW to 20 μW, the signals also broadened by about 15%. In turn, the slope of the resonance, which determined the sensitivity of the magnetic field measurements (see Section 3.4), remained nearly unchanged.

**Figure 4:**
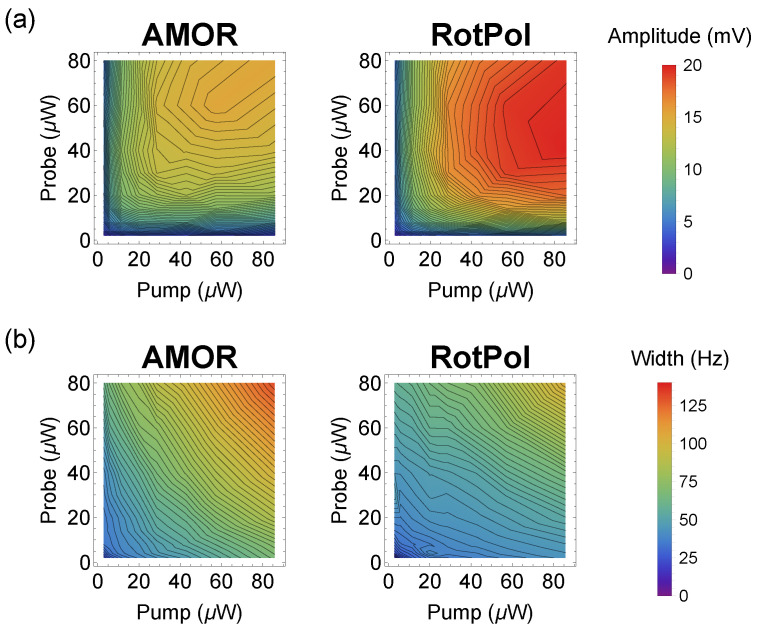
Amplitude (**a**) and width (**b**) of the AMOR (left column) and RotPol (right column) signals (central resonance) versus the pump and probe powers.

To verify whether under different conditions the efficiency of AMOR generation did not exceed that of the optimal RotPol, we measured the amplitude and width of both signals versus the pump and probe powers (Figure 4).

The obtained results show that the amplitude of the resonance measured in the RotPol was always larger than that of the corresponding AMOR resonance. Moreover, for the RotPol, the strongest resonance was observed for a relatively strong pump and not-too-strong probe. At the same time, in the AMOR, the strongest resonance was observed for a weaker pump and stronger probe. The resonance, however, was about 30% weaker than in the RotPol case. Under the same conditions, the width of the observed NMOR resonances was about 30% narrower, though the trends in both techniques were the same (the width monotonically increased with the power of either of the beams).

**Figure 5:**
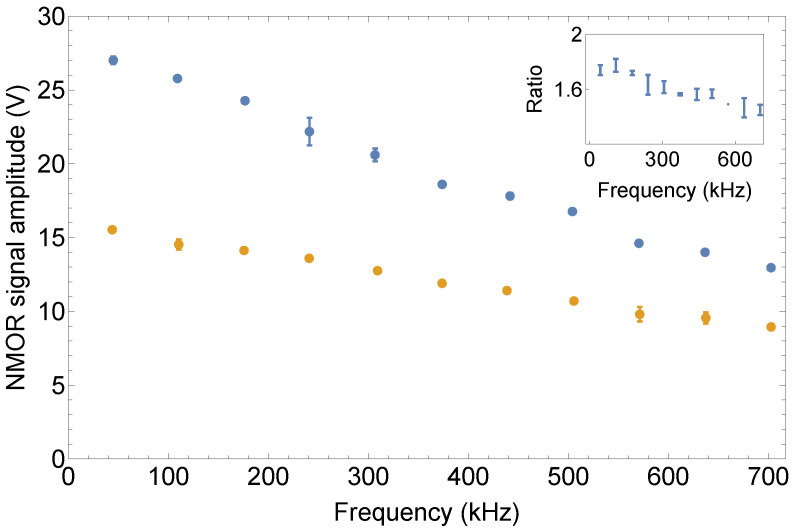
Amplitude of the NMOR signal versus magnetic field measured with RotPol (blue) and AM (orange) lights.

Another interesting aspect of the signals was their dependence on the magnetic field/Larmor frequency. Analysis of this dependence allows one to study the hypothesis of the AOC-induced deterioration of the signal. Figure 5 shows the amplitude of the NMOR signal measured with both the AM and RotPol lights versus the magnetic field.

The results show that the amplitudes of the RotPol resonances were larger than the AMOR ones (about 1.5 times, as shown in the inset to Figure 5). While the amplitude ratio revealed a relatively weak dependence on the leading field, the individual amplitudes depended on the field, where they experienced about a 50% reduction when transitioning from weak to strong fields. This is somewhat surprising in the scope of previous works, where a significantly stronger leading-field dependence was demonstrated [51]. This also indicates that the AOC does not play an important role in the signal deterioration, leaving the question of why such a reduction was observed previously. While the answer to this question may only be speculative, one may expect that the effect was either induced by broadening of the resonance due to an increase in the absolute inhomogeneity of the applied magnetic field [57] or by some filtering effect present in the experimental systems, leading, for example, to the inability to perform 100% modulation (where optical pumping was either less efficient or repumping destroyed the atomic polarization). In the presented studies, this effect was not present due to a different technique of generating the AM light.

### 3.3. Spectral Dependence of the RotPol Signal

To study the spectral dependence of the RotPol signals, the experimental setup was modified by incorporating a separate probe laser (a fraction of the light beam originally used for probing was blocked). This modification allowed for independent control of the tuning of both lasers, which facilitated the search for the optimal signal.

**Figure 6:**
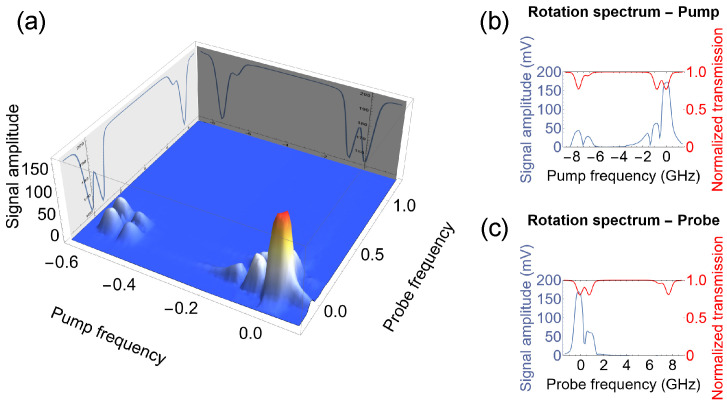
Dependence of the amplitude of the RotPol signal on the tuning of the pump and probe lights. Plot (**a**) shows the signal for a set of various tunings across the D_1_ line. The two plots on the right, (**b**,**c**), show projections of the signal into two planes. Plot (**b**) illustrates the dependence for the probe tuned close to the center of the Doppler-broadened $F=2\to F^{\prime }=1$ transition, with the pump scanned across the D_1_ line. Plot (**c**) presents the corresponding signal when the pump was tuned to the center of the Doppler-broadened $F=2\to F^{\prime }=1$ transition, and the probe was scanned across the same line.

Figure 6 shows the amplitude of the observed signal as a function of the wavelengths of both lasers.

As demonstrated, the strongest signal was observed when the lasers were tuned to the center of the Doppler-broadened $F=2\to F^{\prime }=1$ transition. This suggests that for such tunings, the largest transverse polarization was being generated in the medium and its effect on the probe light polarization was also the strongest. Weaker signals appeared when only one of the lasers was tuned to the $F=2\to F^{\prime }=1$ transition while the other laser was tuned to the same ground but different excited state (the $F=2\to F^{\prime }=2$ transition). Under such conditions, either the magnitude of the generated atomic polarization or its effect on the probe light polarization was weaker. This originated from the difference in value and opposite in sign dipole matrix elements for the two transitions. In addition, two distinct peaks in the rotation were observed for the $F=2\to F^{\prime }=2$ pump tuning (Figure 6b). These peaks mark a reversal in the direction of magneto-optical rotation, marked by the zero crossing (as the plot illustrates the absolute value of the rotation). This phenomenon aligns with behaviors previously observed in various NMOR studies [3]. Finally, the signal was also observed for both beams tuned to the $F=2\to F^{\prime }=2$ transitions, though its amplitude was even smaller compared with previous cases.

The results shown in Figure 6 also indicate the presence of a signal for the pump tuned to the $F=1\to F^{\prime }$ transition. Although polarization rotation under this tuning is observed in both conventional (unmodulated-light) [3] and modulated-light NMOR [45], the situation in question was qualitatively different. In conventional NMOR, the opposite rotation directions of the spins in two ground-state hyperfine levels (nearly opposite gyromagnetic ratios) are irrelevant due to slow precession (the spin relaxation rate is comparable with the Larmor frequency) such that the optical pumping of transverse atomic polarization (transverse alignment) is efficient for both tunings. At higher fields, when either FM or AM light is used, the rotation is indeed opposite. In this case, however, the modulated light comprises both spectral components shifted by $\pm \nu_{m}$, ensuring that one component may be resonant for either tuning (this situation is analogous to nuclear magnetic resonance, where nuclear spins are excited by a component of the oscillating radio-frequency signal that co-rotates with the spins). In the RotPol scenario, where light precesses in a specific direction, the strong dynamic polarization of the medium can only be generated when spins co-precess with the light polarization. In turn, in the other hyperfine level, the light does not induce the transverse atomic polarization, but generates an average static atomic polarization (longitudinal alignment), undetectable with polarization rotation of light propagating in the same direction as the pump. Thereby, the rotation signal should only be observed for a given hyperfine tuning. However, our data show that such a signal was also present for the other transition (Figure 6b). After a careful analysis, it turned out that this signal was not a magnetic-induced signal but rather an artifact that originated from the optical pumping. Specifically, the strong pump optically polarized the atom at any time, which created in this way a weak transverse component of atomic polarization synchronized with its rotation. In this way, the probe experienced modulated properties of the medium, which manifested when the pump was tuned to the other hyperfine state. As this signal does not depend on the magnetic field, it is not interesting from the perspective of magnetometry.

Our analysis showed that the optimal conditions for NMOR signal generation were achieved when the pump and probe had similar tuning. This suggests a possibility of using a single laser for both the pump and the probe.

### 3.4. Sensitivity to Magnetic Fields

The results presented above demonstrate that the amplitude *A* of the NMOR resonance in the RotPol was greater than that observed in the AMOR and that the width γ of the former was narrower than that of the latter. From a practical point of view, the sensitivity of an optical magnetometer is determined by its ability to detect the smallest changes in the resonance position. This means that the sensitivity is given by the slope of the resonance in its central part and the measured noise, which can be written as

(2) $$\delta B=\frac{g\mu_{B}}{\hbar }\times \frac{\gamma }{\mathrm{SNR}},$$

where $\mu_{B}$ is the Bohr magneton, *g* is the Landé factor, *ℏ* is the reduced Planck constant, and SNR is the signal-to-noise ratio. In turn, studying the dependence of the parameters on NMOR resonances allowed us to compare the sensitivity of the RotPol and AMOR magnetometry.

**Figure 7:**
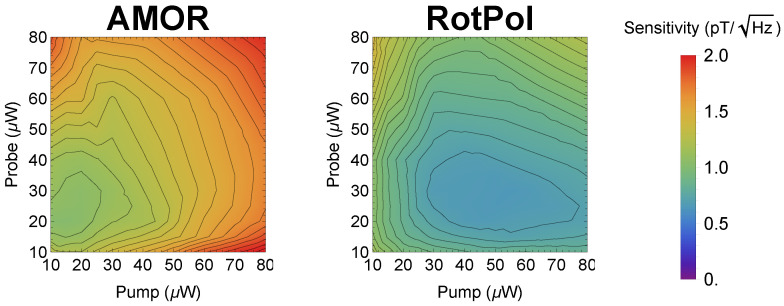
Sensitivity of the stronger (≈30 μT) magnetic field measurements using the AMOR (**left**) and RotPol (**right**) light versus the pump and probe powers. For the measurements, a single laser with a split light beam was used.

Figure 7 presents the comparison of the RotPol and AMOR magnetometry as a function of the pump and probe powers.

The results show that in the RotPol, the optimal sensitivity of 650 fT/Hz^1/2^ was achieved with a pump power of 55 μW and a probe power of about 30 μW. The AMOR achieved an optimal sensitivity of 1.15 pT/Hz^1/2^ with a pump power of 20 μW and a probe power of 25 μW. Although the sensitivity might be somewhat lower compared with other techniques [8,58,59], it is important to note that these measurements were performed in a magnetic field of 100 μT, which exceeds the typical operating conditions of optical magnetometers by at least a factor of 3, and more often by 3–4 orders of magnitude. This particularly distinguished this study from other research.

## 4. Discussion

The presented technique provides the first example of NMOR-based magnetometry applied to fields that significantly exceed Earth’s magnetic field (note that unmodulated-light NMOR was previously studied in such or stronger fields [60,61]). Utilizing such an ultrasensitive technique with a broad dynamic range may be important for various applications. For example, in space exploration, NMOR-based magnetometry can be used for the measurement of interplanetary magnetic fields [62], as well as fields in celestial and planetary environments, enabling, for example, investigations of Jupiter’s magnetosphere [63] or facilitating the search for magnetic anomalies on Mars [64]. Moreover, in materials science, examining the magnetic properties of materials under stronger fields may provide new insights into their structure and magnetic or mechanical defects [65]. Finally, measurements of magnetic fields slightly stronger than Earth’s magnetic field can be beneficial in geophysical navigation by detecting magnetic anomalies associated with specific natural resources or facilitating the identification of subsurface geological structures [21].

A different aspect of the presented research is the potential improvement of the magnetometer performance, e.g., its sensitivity. A specific method for such an improvement is through increasing the concentration of the vapor. As shown in Ref. [8], increasing the medium concentration to approximately one optical depth maximizes the sensitivity of magnetic field measurements. It is clear from Figure 7 that our magnetometer was far from being optimized in this regard, and it is feasible to increase the concentration by more than an order of magnitude. Adopting this strategy could lead to a significant increase in the sensitivity of magnetometric measurements; however, it should be performed in conjunction with monitoring the system performance (e.g., monitoring the width of the NMOR resonance) in order to avoid melting the paraffin, which was our anti-relaxation coating (melting the paraffin would increase the NMOR resonance linewidth by many orders of magnitude.

Finally, it is worth noting the potential to use this RotPol technique in a self-oscillation mode [58,59]. In such a case, the appropriately amplified polarization rotation signal can be fed back as a modulation signal to the polarization rotation system. Since the atoms themselves act as a narrow-band filter, this configuration will favor frequencies associated with the Larmor frequency, and spin precession at this frequency will be amplified. This will lead to the precession of spins at the Larmor frequency and successive automatic tracking of the field changes. We have already built such a system and tested its operation [66].

A challenge with the described solution is developing a simple and reliable system for generating rotating polarization. While a bulk setup using a Mach–Zehnder interferometer combined with acousto-optic modulators is conceptually straightforward, its implementation and reliability pose certain technical issues. Thus, an attractive approach could involve creating such a system using monolithic optoelectronic systems, incorporating the system onto a single chip [67,68]. Integrating an interferometer with phase-shifting elements could potentially address some of the aforementioned technical challenges. Alternatively, electro-optic modulators could be employed to manipulate light polarization. In this case, an electric field would dictate the spatial orientation of the linear polarization. However, the inability to continuously increase the electric field value would necessitate either “resetting” the field—causing the light’s phase to advance monotonically only within a certain angular range and then abruptly unwrapping the rotation—or developing a solution in which combining the electric fields across one or more electro-optic crystals results in continuous light rotation. So far, though, none of these solutions have been fully developed.

## 5. Summary and Conclusions

To the best of our knowledge, this article presents, for the first time ever, an application of the continuously rotating linear polarization in any atomic physics experiment (or in any experiment for that matter). Specifically, we used such light to analyze optical pumping in the context of NMOR, which synchronized the light polarization rotation with the Larmor frequency of the atomic spins, which occurs when a dynamically precessing atomic polarization of significant magnitude is generated within the medium. This transverse anisotropy strongly modulates the properties of the independent, unmodulated linearly polarized light traversing the medium, enabling the detection of nonlinear magneto-optical rotation signals with large amplitudes. This large amplitude polarization signal translates to a high sensitivity for this method compared with other NMOR techniques that utilize modulated light. The method itself demonstrated the capability to measure magnetic fields across a wide range up to fields nearly three times greater than Earth’s magnetic field, as the pumping conditions do not depend on the light precession frequency. Thus, the method presents a unique combination of very high magnetometric sensitivity—which, as discussed, can be increased by up to threefold—and an exceptionally large dynamic range. This makes our technique potentially useful for precise magnetic field measurements across a broad dynamic range in future applications.

The presented results also allowed us to verify the hypothesis regarding the role of the alignment-to-orientation conversion in the reduction of the NMOR signal amplitude with increasing magnetic field strength, as previously observed in the literature. Our study demonstrated that in scenarios where the modulation parameters of the light, including its duty cycle and amplitude, do not degrade with increasing magnetic fields, the observed reduction in the amplitude is relatively small (approximately 40%) and similar for both the RotPol and AMOR techniques. This evidence suggests that the AOC was not responsible for the amplitude reduction. Instead, the decline was likely due to the nonlinear splitting of the resonances and the gradual decoupling of hyperfine interactions by the magnetic field, as well as the emergence of field inhomogeneities.

As discussed above, the RotPol method offers promising opportunities for practical applications in “in-field” magnetometry. To fully realize this potential, two important technical challenges must be effectively addressed. The first is to mitigate the effect of mechanical vibrations, which is specific to the RotPol approach, while the second challenge consists of reducing the effect of environmental (electro)magnetic perturbations. As the second problem is common to all “in-field” magnetometry techniques, one can address it by adopting a conventional approach employed in such cases. For example, it can be alleviated by operating in the so-called gradiometric mode, where two magnetometers are used [15,35,37,40]. In this configuration, one magnetometer is positioned near the source of a magnetic field of interest to measure both the source magnetic field and the environmental magnetic field/noise, while another magnetometer, situated farther from the source, measures only the environmental field. Assuming the environmental magnetic field varies over much larger distances than the distance between the two magnetometers and that the source field changes much more quickly, the subtraction of the fields measured by the magnetometers allows for more precise determination of the field from the source. Depending on the specific implementation and environmental conditions, this method could enhance the sensitivity of weak signal detection by factors ranging from several to several thousand times.

In RotPol magnetometry, the problem of mechanical noise predominantly originates from the application of an optical interferometer for the generation of light with rotating polarization. Since interferometers are designed to sense the phase difference between two light beams, variations in their optical path lengths strongly affect the interference. Recognizing this challenge, our RotPol system incorporates an additional component to monitor and control the phase of rotation [54]. Specifically, the rotation phase is continuously monitored with a photodetector located behind a polarizer placed after the vapor cell in the pump beam path (the detector records a harmonic signal). If there is a change in the optical path in one arm, it leads to a phase change in the rotation, affecting the detector signal. This signal is then compared with the low-frequency polarization-rotating signal used in the modulator, shifting the RF signal and driving one of the AOMs. If a phase shift is detected between the two signals, an electronic signal delay line implemented in the electronics driving the other AOM is used to compensate for the phase change. As shown in Ref. [54], this method significantly reduces the phase drifts originating from turbulent airflow. It is believed that the same system, with sufficient bandwidth, will also mitigate the influence of mechanical vibrations on polarization rotation stability, thereby enhancing the sensitivity of the magnetometric measurements.

## Figures and Tables

**Table 1:** Amplitude, width, and slope of the NMOR signals observed with RotPol and AM lights. The AM data are shown for two average light powers: one corresponding to the same peak intensity as in the RotPol case and the other to the same average light power (a factor of 2 difference between the cases).

Type	Pump Power	Amplitude	Width	Slope
	(μW)	(V)	(Hz)	(mV/Hz)
RotPol	10	1.947 (26)	27.17 (81)	71.6 (3.1)
AM	10	0.8952 (88)	35.99 (80)	24.87 (80)
AM	20	1.1250 (82)	41.68 (60)	26.99 (59)

## Data Availability

The data used in this study will be made available upon reasonable request. Additionally, they will be deposited in a Jagiellonian University data repository where they will receive a DOI and will be publicly accessible along with explanations regarding their collection and processing.

## Abbreviations

The following abbreviations are used in this manuscript:

OAM	Optical atomic magnetometer
NMOR	Nonlinear magneto-optical rotation
AMOR	Amplitude-modulated nonlinear magneto-optical rotation
RotPol	Rotating polarization
AOC	Alignment-to-orientation conversion
AOM	Acousto-optical modulator
BS	Beam splitter
PBS	Polarization beam splitter
